# Pax7 is back

**DOI:** 10.1186/s13395-014-0024-4

**Published:** 2014-12-14

**Authors:** Andrew S Brack

**Affiliations:** Center for Regenerative Medicine, Massachusetts General Hospital, Boston, MA 02114 USA; Harvard Stem Cell Institute (HSCI), Boston, MA 02114 USA; Harvard Medical School, Boston, MA 02115 USA

**Keywords:** Pax7, Satellite cell, Skeletal muscle, Regeneration, Lineage tracing, Cre

## Abstract

Two recent studies have reinvigorated the conversation regarding the role of Pax7 in adult satellite. Studies by Gunther *et al* (Cell Stem Cell **13:**590–601, 2013) and Von Maltzhen *et al* (Proc Natl Acad Sci U S A **110:**16474–16479) show that Pax7 is critical for adult satellite cell function and their contribution to muscle repair. Previously, Lepper *et al* (Nature **460:**627–631, 2009) demonstrated that Pax7 was dispensable for adult muscle repair. In this commentary I have summarized the results from these studies, focusing on the differences in experimental paradigms that led the authors to different conclusions. I also take this opportunity to discuss the potential limitations and hurdles of Cre-lox technology that are responsible for the discrepant results.

## Background

Adult skeletal muscle has a remarkable regenerative capacity due to a rare population of cells termed satellite cells that are wedged between the basal lamina and sarcolemma, interspersed along each adult muscle fiber [1]. Since the original characterization of Pax7 as a specific marker of adult satellite cells there has been a concerted effort among skeletal muscle biologists to ascertain its role in satellite cell function [2]. Germline mutants and *in vitro* knockdown studies have all convincingly shown a key role for Pax7 in survival and proliferative potential of satellite cells and their committed progenitors, called myoblasts [2-5]. Until recently, a definitive demonstration for the functional role of Pax7 in adult satellite cells *in vivo* was lacking. Over the past 5 years, three laboratories have examined the requirement for Pax7 in adult satellite cells using an inducible mouse strain that allowed Pax7 to be normally expressed during development and subsequently deleted in adult mice [6-8]. While the mouse genetics used by the labs were identical, the regimens employed to delete Pax7 were not, resulting in very different outcomes and interpretations.

## Main text

Since the first description of gene targeted mice in 1989, which led to the Nobel Prize being given to Cappeci, Evans, and Smithies in 2007, there has been an exponential use of genetic mutant mouse models to study mammalian gene function. Today, sophisticated genetic strategies that allow biologists to delete a gene of interest from a cell and its descendants in a temporally controlled manner have now become almost commonplace. The tamoxifen (TMX) inducible Cre lox system is used most frequently in mouse models. A modifed Cre recombinase fused with an estrogen receptor is expressed under the control of a specific promoter, selected due to its tissue or cell type restricted expression. Active Cre will recombine loxP sites, engineered to flank a gene of interest, resulting in a null allele. This approach has been used to indelibly mark and track a cell and its descendants. In such cases, loxP sites flank a stop sequence upstream of a fluorescent gene reporter inserted into the accessible ROSA26 locus.

The muscle scientific community is fortunate to have has four different inducible Pax7 Cre alleles at its disposal [6,7,9,10]. All four lines are knockin alleles, three containing an internal ribsome entry site (IRES) to retain Pax7 translation [7,9,10], and one with increased Cre sensitivity (Cre^ERT2^) [10].

**Figure 1:**
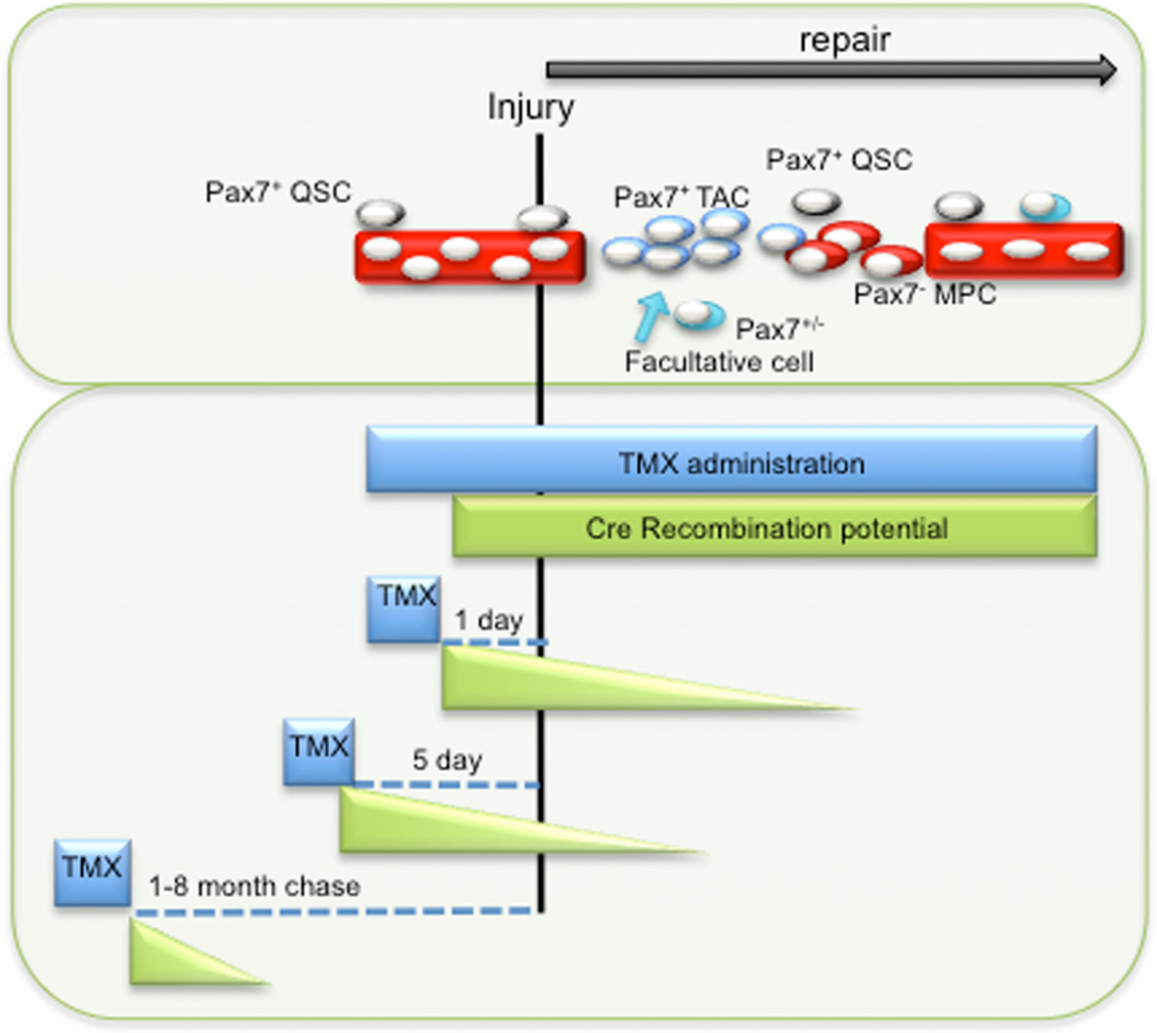
**Strategies to target Pax7 cells using inducible Cre recombination and its implications for defining the cellular dynamics during muscle regeneration.** Upper box: In response to injury, Pax7+ satellite cells exit from quiescence (QSC) (grey cells) to produce Pax7+ rapidly dividing transit amplifying progenitor cells (TAC, blue) that differentiate (myogenic progenitor cell, MPC), coupled to a decrease in Pax7 levels (-) (red) before forming new muscle. Proliferating Pax7+ SCs return to quiescence (grey cells) and reoccupy the niche. After injury, facultative cells (cyan) that express Pax7 transcript transiently (+/-) enter the muscle and contribute to the repair process. Lower box: Different TMX strategies (blue box) employed to target Pax7+ cells, before (followed by a chase: dashed line) and during injury. TMX half-life is estimated at around 10 days *in vivo*, therefore recombination of Pax7+ cells is possible following the TMX administration regimen (green box and triangle). If TMX is administered shortly before or during injury, Cre has the potential to recombine escaper Pax7+ QSCs as they proliferate (TAC). Other facultative cells that transiently express Pax7 transcript as part of the regeneration process and SCs that return back to quiescence can also be targeted. In all of these aforementioned scenarios, deletion of Pax7 gene if functionally important, will exacerbate the muscle phenotype, but at the expense of identifying the cell state that requires the gene. Targeting with TMX months prior to injury allows a definitive conclusion to be made against the QSC. This is critical since Pax7 levels differ across the QSC compartment and change dynamically during lineage progression.

To test the requirement for Pax7 in adult satellite cells, that is, delete Pax7 from Pax7 expressing satellite cells, the laboratories of Fan, Rudnicki, and Braun used a mouse strain that harbored the same Pax7-loxP and TMX-inducible Pax7Cre allele, developed by Lepper *et al*. TMX was administered to adult mice, either immediately prior to injury, 5 days prior to injury, immediately prior to injury and during repair, and prior to injury with a long chase period, in the range of 1 to 8 months (see Figure 1). The results clearly show that satellite cell function and regeneration capacity is significantly impacted when TMX is chased for long periods prior to injury or administered during repair. In response to muscle injury, there was a reduction in the number of Pax7-expressing cells, progressive loss of muscle tissue, increased myofibroblasts, and an accumulation of fatty infiltrate [7,8]. Importantly, targeting Pax7 in satellite cells and their progeny in a cell culture model reduced satellite cell proliferative capacity, due to precocious differentiation. In stark contrast, regeneration was unaffected if a short chase (5 days) followed TMX administration prior to muscle injury [6]. Despite the normal regenerative output, assessment of recombination based on β-galactosidase (β-gal) activity driven off a ROSA26.LacZ reporter revealed a robust contribution from recombined Pax7+ satellite cells to regenerating muscle fibers. In support of their *in vivo* findings, Lepper *et al*. reported that freshly isolated β-gal + satellite cells appeared to be capable of normal replication and differentiation in cultured conditions.

Two compounding factors are likely responsible for the different outcomes of Pax7 deletion on muscle repair: (1) limitations in Cre lox technology, and (2) the insatiable contribution of optimally functioning satellite cells to the muscle repair process. Successful application of inducible Cre lox technology relies on many individual elements working optimally, including uniform Cre activation in all cells within a target population, efficient gene recombination, and loss of the gene product (protein). In the presence of suboptimal gene targeting, resulting cellular phenotypes can be misinterpreted as evidence for redundant gene function.

The potency of Pax7 expressing satellite cells to proliferate and repair tissue is unquestionable. Therefore, a rare non-recombined satellite cell with presumed normal function (‘escaper’ cell) will propagate under the stimulus of injury and contribute robustly to tissue repair. Lepper *et al.* demonstrated extensive contribution of recombined (β-gal+) Pax7 derived cells to regenerated muscle fibers. However, strict conclusions on recombination efficiency cannot be drawn in a multinucleated syncytium such as skeletal muscle; it cannot be ruled out that ‘escaper’ cells contributed extensively to regenerated muscle fibers.

To analyze recombination at the level of Pax7, Lepper *et al.* isolated whole muscle or small numbers of cells from muscle tissue, with both methods showing Pax7 was not expressed after TMX administration. In contrast, Van Maltzhan *et al.* demonstrated that when a population of TMX treated satellite cells is contaminated with rare escaper cells, this small fraction of wild type cells could proliferate and out-compete the majority of recombined cells, achieving a repopulation of the cell pool with wild type function. This warns against performing analysis on low cell numbers and from whole tissue when making interpretations about rare cell populations. Moreover, during the course of our experiments using mice harboring inducible Cre alleles, we have noticed variable recombination efficiency between mice, even within the same litter and of the same gender. Therefore, assessment of recombination in every experimental animal is strongly advised.

The persistence of active TMX increases the likelihood that a gene locus will undergo successful recombination. *In vivo* estimates of TMX half-life is 7 to 11 days [11,12], therefore, after a 24 h chase or TMX administration during repair, as performed by Van Maltzahn *et al.* and Gunther *et al.*, residual TMX and hence Cre activity could act on proliferating satellite cells and their downstream Pax7 expressing progeny, thus increasing the likelihood of recombination in escaper cells. This may explain, in part, why chasing TMX for 1 day instead of 5 days prior to injury led to a regenerative decline. In support of the escaper cell hypothesis, proliferative compensation of satellite cells was prevented after mice received TMX in their chow during the regeneration process [8].

The stability of the targeted protein also needs to be considered when designing a TMX strategy. If protein turnover is longer than the length of chase period, many cells will have residual protein at sufficient levels to preserve normal function. Using the Lepper *et al.* allele and chasing TMX for months instead of weeks, Gunther *et al.* observed a requirement for Pax7 in satellite cell maintenance and function during repair.

In addition, Gunther *et al.* examined a different inducible Pax7Cre^ER^ and Pax7 loxP allele in which Pax7 protein was more rapidly degraded. A shorter TMX chase was required to deplete the satellite cell pool and lose muscle regenerative capacity. Therefore, retention of Pax7 biological activity altered the kinetics of stem cell diminution. Based on the sum of the data, it is appropriate to conclude that Pax7 is critical for normal adult satellite cell function and in its absence skeletal muscle repair is severely compromised.

While it is becoming clear that high levels of recombination should not be assumed and strategies to maximize recombination are desired, caution is also advised. It was recently demonstrated that TMX promoted apoptosis in the intestine and biased the contribution of one stem cell subset over another without impacting tissue morphology [13]. In the same vein, long-term TMX delivered to mice in their chow, while increasing recombination efficiency may have unintentional consequences that influence satellite cell contributions. Neither Gunther *et al.* nor Maltzahn *et al.* reported detrimental effects of long-term TMX on muscle regeneration, however its effects on the satellite cell population and muscle repair warrants further scrutiny. Therefore, it is imperative that non-specific effects of TMX administration and Cre lox recombination that could indirectly alter cell function are considered and minimized.

On one hand, long-term administration of TMX leads to greater deletion efficiency but at the same time masks whether the cellular phenotype is borne from quiescent cells specifically and/or their Pax7+ progeny. This issue is particularly relevant if downstream progenitors have different levels of Pax7 and are biased towards differentiation or self-renewal, as reported [14]. In such a scenario, cells expressing low levels of Pax7 are less likely recombined and retain wild type function. In addition, it has become apparent that muscle resident non-satellite cells participate in muscle repair, of which some cell types may transiently activate the Pax7 promoter [15-17]. If facultative cells are identified in adult muscle that express and require Pax7 for muscle contribution, administration of TMX immediately before or during repair will induce recombination in these cells. In contrast, administering TMX prior to injury and with adequate chase, would avoid recombination of these transient Pax7 expressers, presumably retaining their normal function and contribution to muscle tissue repair (see Figure 1). Therefore, prolonged TMX administration will increase the likelihood of targeting transient cell populations. Technologies that allow inducible and reversible gene deletion strategies such as doxycycline inducible reverse tetracycline-controlled transactivator system (rtTA) will allow examination of the state specific role of Pax7 in satellite cells as they progress along their lineage or other Pax7 transiently expressed populations as they contribute to muscle repair.

Due to the caveats of TMX stability, its long-term administration for effective gene recombination and the dynamic cell populations within a regenerating muscle, it would be useful to determine how long TMX can retain recombining activity of satellite cells *in vivo*. To this end, one could administer TMX to wild type mice that are chased for varying lengths of time and subsequently injected with satellite cells harboring Pax7Cre^ER^-loxP ROSA26 reporter alleles into pre-injured muscle. The presence of reporter expression in the regenerated muscle would provide an estimate of TMX recombining half life. These results would help in the design of experiments that maximize recombination efficiency in satellite cells prior to injury, or targeting transit-amplifying cells (TAC) cells and facultative cells during injury (see Figure 1).

As the chapter on Pax7’s requirement for adult satellite cell function comes to a close, what is next for Pax7 and the satellite cell? The phenotypic differences between the work of Lepper *et al.* and that of Van Malzhen *et al.* and Gunther *et al.* can be distilled to the presence or absence of ‘escaper’ cells. Which begs the question: are Pax7+ escaper cells a unique population or a stochastic anomaly? Escape from recombination can arise from many factors, some technical, such as suboptimal TMX, or biological variances, including chromatin compaction and gene methylation or promoter activity. I would argue it unlikely that TMX dosage played a role. Comparisons between the different administration strategies employed by Lepper *et al.*, Van Maltzen *et al.*, and Gunther *et al.* strongly suggest that cell cycle state (quiescence versus proliferating) may impose resistance to recombination. It is important to keep in mind that Lepper *et al.* observed substantial recombination of the ROSA26 locus in both neonatal and adult muscle, however, only in neonatal muscle, which comprises mainly cycling Pax7+ cells, did a phenotype manifest.

A characteristic morphological feature of quiescent stem cells is their tightly packed heterochromatic state. Is it possible that escaper cells have more compact chromatin, which is relieved during the cell cycle? Gunther *et al.* highlighted an unappreciated role for Pax7 in maintenance of the heterochromatic state; after Pax7 deletion, satellite cells took on a euchromatic morphology, which preceded their numeric and functional loss. It will be important to resolve whether subsets of satellite cells have more densely-packed chromatin and whether deregulation of chromatin architecture via Pax7-dependent and independent mechanisms lead to changes in satellite cell function.

Gene methylation is a significant roadblock for Cre mediated recombination [18]. Therefore, inaccessible loci will more likely avoid recombination, leading to the retention of normal gene function. To circumvent this limitation, due to its open configuration, the ROSA26 locus is often targeted to insert Cre mediated reporters. While this is a reliable approach for lineage tracing studies, it should be used cautiously as a proxy for gene deletion. Methylation status of a target gene at the time of TMX administration will impact the accuracy with which the ubiquitous reporter reflects deletion of a specific gene. Therefore, direct measurement of recombination of the targeted gene locus or protein within the cell of interest is critical. In Table 1 I have outlined TMX strategies for optimal gene recombination, measuring recombination efficiency and testing escaper cell contribution.

**Table 1:** **Strategies for effective gene recombination in adult satellite cells and their progeny during muscle repair**

**TMX delivery**	**Recovery period**	**Gene deletion analysis**	**Escaper cell analysis**
TMX injection at 3 mg/day for 5 consecutive days^a^	Allow >15 days of TMX chase prior to muscle injury to target quiescent SCs and minimize lingering TMX activity	Isolate purified SCs by FACS, or from single fibers from each experimental animal^b^	Measure gene or protein levels over time^c^
Five daily injections prior to injury followed by continuous TMX feeding delivered in chow (1 mg TMX per day)	**Pros:** Temporal identity of cell type and state	Measurement of protein by western blot or immunohistochemistry	***In vitro*** **:** Measure gene/protein immediately after isolation and after approximately 10 days in culture
	Minimize recombination of transient cell populations	Quantify DNA directly at the gene locus using site-specific primers flanking the *loxP* sites	***In vivo*** **:** Measure gene/protein from SCs isolated from uninjured and regenerated muscle (>30 days after injury)
	**Cons:** Escaper cell contribution		
	No recovery period		
	**Pros:** Maximize targeting of both stable and transient cell populations		
	Minimize escaper cell contribution		
	**Cons:** Lose cell type and state resolution		

^a^Recommendation based on 30-gram adult mouse.

^b^Due to variable recombination efficiency between mice, assessment of recombination in every mouse is strongly advised. In addition, I caution the use of R26R-based fluorescent reporters as a surrogate to assess recombination of a gene of interest.

^c^In the presence of escaper cells, gene/protein levels of the targeted allele will increase over time if the recombined gene has a loss-of-function phenotype. If recombination results in a gain-of-function mutant or acts redundantly, then gene/protein levels will decrease or not change, respectively. In the absence of escaper cells, the gene/protein of interest will not be detectable.

## Conclusions

In closing, the studies from Gunther *et al.* and Van Maltzen *et al.* wrap up the debate about the importance of Pax7 in adult muscle regeneration. However, the mechanisms that preserve satellite cell homeostasis in the absence of injury, the role of Pax7 as a modifier of chromatin architecture, and the requirement of Pax7 in muscle resident facultative cells are not yet solved. In addition, while we critically try to minimize, or at least control for, the limitations of inducible Cre lox technology, it is also worth considering the possibility that such limitations, if understood, may be exploited to help define cellular and molecular heterogeneity within the satellite cell compartment.
